# Rapid Preparation of Flame-Retardant Coatings Using Polyurethane Emulsion Mixed with Inorganic Fillers

**DOI:** 10.3390/polym15030754

**Published:** 2023-02-02

**Authors:** Yaokai Wang, Jinfang Liu, Xu Pan, Min Zhao, Jianfu Zhang

**Affiliations:** 1 School of Chemistry and Environmental Engineering, Changchun University of Science and Technology, Changchun 130022, China; 2 Jilin Provincial Science and Technology Innovation Center of Optical Materials and Chemistry, Changchun 130022, China

**Keywords:** water-based polyurethane, flame retardant, demulsification-induced fast solidification, flame-retardant coating

## Abstract

The traditional aqueous flame-retardant coating faces the problem of slow solvent evaporation rate in the preparation process. It is an urgent problem to ensure that the function of the membrane is not destroyed while accelerating the solvent volatilization. Herein, we fabricated films on the metal substrate surface by a totally novel method: demulsification-induced fast solidification to rapidly obtain the flame-retardant coating. The environmentally friendly flame retardants aluminum hydroxide and red phosphorus were mixed with the commercial water-based polyurethane 906 emulsion to explore the optimal mixing ratio, where the adhesion of the flame-retardant reached the Grade 3 standard, the sample remained intact after the 100 cm drop hammer test and the limiting oxygen index value reached 30.4%. In addition, compared with the traditional process, this method, with the advantages of rapidly drying, environmentally friendly, uniformly prepared coatings on the surface of any shape substrates, as well as accurate and controllable coating thickness, can be widely applied in the flame-retardant field.

## 1. Introduction

Flame-retardant coatings are protective coatings that can inhibit or delay the combustion of materials; moreover, they can effectively reduce the probability of fire occurrence and retard the spread of fire, significantly enhancing the fire resistance and heat resistance of materials [1,2]. Steel structure houses, metal cables, electric and instrument enclosures, and other metal materials have certain fire-protection needs [3,4]. Flame-retardant coatings can greatly improve the fire resistance of steel structures and prolong the time for which they remain rigid in fires and other high-temperature environments [5]. Additionally, the flame-retardant coating can considerably reduce the risk of heat accumulation and fire in cables and electric and instrument enclosures under extreme working conditions [6].

The current methods to prepare flame-retardant coatings include spraying and brushing [7,8,9]. Spraying and brushing have good coating capability for flat and regular surfaces, but they do not effectively produce uniform coating for objects with complex and irregular surfaces and internal complex spatial structures. Organic paints can be rapidly and conveniently applied and have relatively short drying times; however, they have a high content of volatile organic compounds (VOCs), resulting in severe environmental pollution [10,11,12,13]. The use of water-based paints can mitigate the pollution problem, but the large latent heat of water vaporization results in an excessively long drying time, which is not conducive to rapid large-scale production. Although heating can accelerate drying, it will increase the application cost, and some materials cannot be heated. Therefore, conventional coating methods and paint types feature significant defects.

Flame retardants are classified into reactive and additive types according to their addition methods [14,15]. Reactive flame retardants are synthesized through the grafting of small flame-retardant molecules, such as halogenated phosphates and 2-chloroethanol, as functional groups to the main or side chain of polymer materials. Their advantages include small impacts on the physical and mechanical properties and long-lasting flame retardancy properties; however, increasing the amount of reactive flame retardant added to substrates is difficult, the preparation process is cumbersome, and the manufacturing cost is high, limiting the application of reactive flame retardants. Reactive flame retardants are a popular research area but need further exploration for industrial use [16]. Additive flame retardants are synthesized through the direct physical blending of the flame retardant and the material to improve the flame retardancy properties of the material. Their advantages include the possibility of adding a high amount of flame retardants to the materials, simplicity of the preparation method, and low cost [17]. However, excessive addition will affect the physical and mechanical properties of the material. Increasing the flame retardant content of a material can improve its flame retardancy properties. Therefore, finding a balance between stability and flame retardancy is vital [18,19].

Additive flame retardants can be divided into inorganic and organic flame retardants. The representative materials of inorganic flame retardants include aluminum hydroxide (ATH), red phosphorus (RP), phosphoric acid, antimony compounds, borides, and magnesium compounds, while those of organic flame retardants include phosphorus-based compounds (e.g., phosphate esters, ammonium polyphosphate), halogens (e.g., bromide, chloride), and nitrogen-based compounds (e.g., dicyandiamide, ammonium sulfamate) [20,21,22]. The flame retardant mechanism usually involves reducing the temperature via heat absorption, reducing the content of oxygen and combustible gas via the release of non-combustible gas, forming a protective film to isolate the material from oxygen, and capturing free radicals to inhibit the combustion process [23].

Presently, environmental protection issues have received increasing attention. Organic paints and toxic flame retardants are gradually being eliminated owing to pollution problems. Water-based paints and environmentally friendly flame retardants are increasingly being developed. However, the long drying cycle of water-based paints cannot be ignored. Similarly, owing to environmental reasons, halogen-free inorganic additive flame retardants tend to be preferred; however, achieving a balance between the amount of flame retardants added and material stability is challenging. To solve the above problems, this study adopted demulsification-induced fast solidification (DIFS) [24] and used water-based polyurethane paints and environmentally friendly flame retardants, namely microencapsulated RP and ATH, to prepare flame-retardant coatings [25,26]. The DIFS method can effectively solve the shortcoming of lengthy application period associated with water-based paint and shorten the coating preparation time. RP and ATH are good environmentally friendly flame retardants. By exploring the performance, flame retardancy, and stability of the flame-retardant coating under different flame retardant–paint mixing ratios, we determined the optimum mixing ratio and prepared the flame-retardant coating.

## 2. Materials and Methods

### 2.1. Materials

The RE–906 polyurethane/acrylate (PUA) tackifier emulsion was purchased from Sihui Bangdeli Chemical Co., Ltd., Sihui, China; microencapsulated RP was purchased from Qingyuan City Yicheng Flame Retardant Materials Co., Ltd., Qingyuan, China; ATH was purchased from Shandong Taixing Advanced Materials Co., Ltd., Rizhao, China; commercially available zinc sheets with a thickness of 0.1 mm and galvanized iron sheets with a thickness of 0.2 mm were selected as metal sheets. Zinc metal sheets were used because it was easy to obtain large-area sheets and the galvanized surface could be used directly.

### 2.2. Measurement Method

The viscosity of the emulsion mixed with the flame retardant was measured under different mixing ratios using a rotational viscometer. The limiting oxygen index (LOI) of the material was determined according to the standard GBT2406.2–2009, and the sample size was 130 mm × 10 mm. The top surface ignition method was employed, and the flammability status was determined according to the following criterion: combustion continued for 180 s or the sample was burned downward for 50 mm; otherwise, the material was non-flammable (Figure S1c). According to the standard GB 1732–1993, a paint film impact tester was used to test the sample. The size of the prepared sample was determined according to the standard that the impact point to the edge of the paint film was >15 mm. Under the impact condition at the corresponding height, the absence of cracks on the paint film indicates that the standard was met at this height condition (Figure S1a). In accordance with the standard GB 1720–1979, the adhesion performance of the coating was characterized using an electric paint film adhesion tester. The standard size of the radius of gyration on the needle tip of the tester was 5.25 mm. Circles with a length of 75 ± 5 mm were drawn on the paint surface, and the sample was compared with the standard diagram. The paint adhesion capability was classified into seven grades: Grades 1 to 7. If >70% of the paint surface in the corresponding area of the sample was intact, the sample met the standard of the corresponding grade (Figure S1b). Table 1 shows the details of the instruments used in the experiment.

## 3. Results and Discussion

### 3.1. Selection of Flame Retardant Type and Mixing Ratio

**Figure 1:**
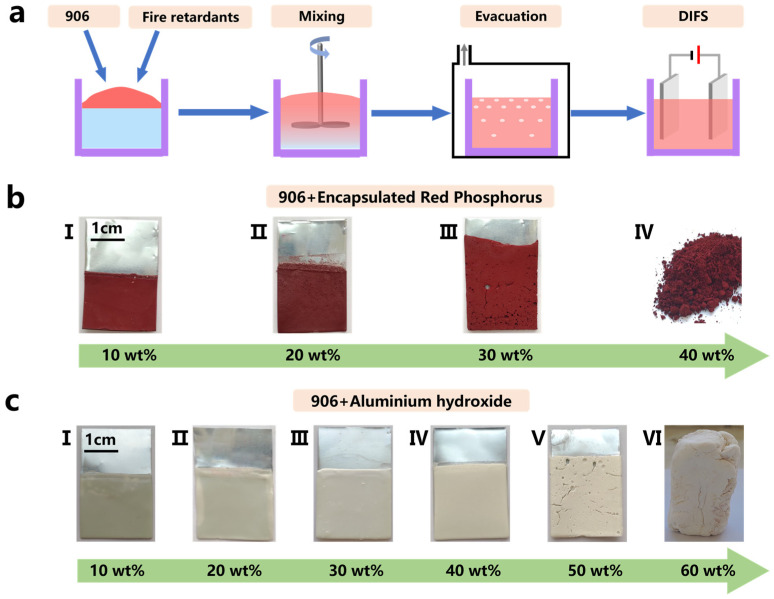
(**a**) Flow chart of the preparation of flame-retardant coating on the metal surface via the DIFS method; (**b**) the flame-retardant coating prepared through the mixing of RP and ATH at different proportions; (**c**) the mixed state.

The flame-retardant paint was prepared through the addition of a flame retardant to a polymer substrate. The RE–906 PUA tackifier emulsion was selected as the polymer substrate because the emulsion has been industrially prepared on a large scale, and purchasing large quantities can substantially reduce preparation costs. The selected flame retardants were RP and ATH. As shown in Figure 1, flame retardants were added to the 906 emulsion, and both materials were mixed according to different mass percentages to determine the optimum mixing ratio. At a too-high viscosity of the mixed emulsion, the internal bubbles could not be self-eliminated, and DIFS-based demulsification and film formation could cause voids and depressions on the coating surface. To ensure the quality of film formation, it was necessary to pump down to create a negative pressure in a closed container, to eliminate the bubbles generated during the mixing process. The time to maintain the negative pressure was adjusted according to the viscosity of the mixed emulsion and the concentration of bubbles, to avoid deteriorating the coating morphology. The 906 emulsion–flame retardant mixture was introduced into a beaker, and zinc sheets were used as the anode and cathode. The area of the electrode sheet was selected as 2 cm × 3 cm, and the depth of the electrode sheet immersed in the emulsion was 2 cm. The sheets were placed parallel to each other at a distance of 1 cm. A voltage of 2 V was stably applied by a DC-regulated power supply, and the time to maintain the voltage in the film-formation stage was uniformly selected as 10 min. After the power was turned off, the cathode sheet was taken out, the emulsion attached to the surface was slowly rinsed with water, and finally, a flame-retardant coating was obtained on the metal surface. The initial flame retardant (RP, ATH) mixing ratio was 10 wt.%, and the mixing ratio was increased at intervals of 10 wt.% to test the film-forming properties until the film could not be formed. The results are shown in Figure 1b,c. The maximum RP and ATH concentrations were 20 wt.% and 40 wt.%, respectively. Under the above concentrations, the flame-retardant coating was smooth, without depressions, and formed a good DIFS film. A continuous increase in the concentration led to defects in the coating. Excessive solid content reduced the overall viscosity and eventually led to the failure of direct solidification in the emulsion, so that DIFS could not proceed, and the film could not be formed (Figure 1b IV and Figure 1c VI).

### 3.2. Coating Growth Curve of One-Component Flame Retardants

**Figure 2:**
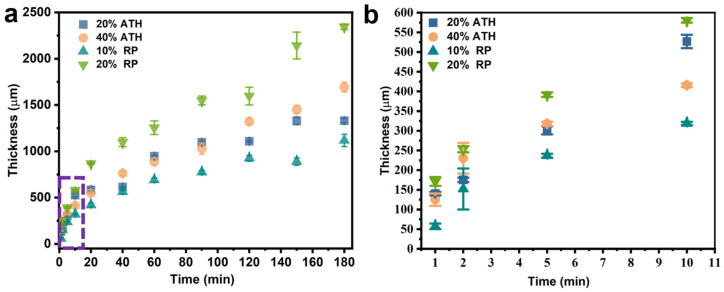
(**a**) The relationship between film thickness and preparation time of the emulsion with different flame retardant concentrations at 2 V; (**b**) the relationship between film thickness and preparation time in the first 10 min.

During film formation via the DIFS method, metal ions are produced on the surface of the anode metal sheet when a voltage is applied, and under the action of ions, the emulsion is demulsified and deposited on the metal surface to form a coating. With the continuous application of voltage, ions are continuously generated and diffused outward, so that the emulsion is continuously deposited on the surface. A zinc sheet with an area of 2 cm × 3 cm was used as an electrode, a voltage of 2 V was applied at a distance of 1 cm, and zinc ions (Zn^2+^) were generated on the zinc sheet surface so that the emulsion continuously produced a paint film on the surface. The relationship between the thickness of the flame-retardant coating and time was studied. With increasing power-on time, the coating thickness increased, but the increase rate reduced over time (Figure 2a). It is speculated that as the emulsion deposition progressed, the diffusion resistance of zinc ions gradually increased, resulting in a gradual decrease in the increase rate of the coating thickness. The coating thickness on the surface of most metal devices was within 500 μm, and the thickness of the decorative coating was roughly between 100–300 μm. For the different concentrations of flame retardant, the DIFS method could be used to prepare a coating with a thickness of 310–570 μm within 10 min (Figure 2b), indicating that a coating with the required thickness can be prepared through DIFS within a short time.

Moreover, the type and concentration of different flame retardants affected the thickness of the flame-retardant coating. The coating thickness increased with increasing flame retardant concentration. Different coatings were prepared using the same proportions of RP and ATH for the same period, and the RP-based flame-retardant coating exhibited a higher thickness than the ATH-based coating.

### 3.3. Mechanical Properties and Flame Retardant Properties of One-Component Flame Retardants

The mechanical properties of the flame-retardant coating were tested, and a flame-retardant coating with an area of 14 cm × 3 cm was prepared on the zinc sheet. The adhesion performance, impact resistance, and LOI values of the samples were measured according to the GB 1720-1979, GB 1732–1993, and GBT2406.2–2009 standards, respectively. The coating prepared using only ATH as the flame retardant exhibited good anti-impact performance, and featured no cracks after the 100 cm drop hammer test. The coating prepared with 20 wt.% ATH exhibited an LOI value of 29.90%, showing good flame retardancy properties. The coating exhibited low adhesion, as the whole coating was peeled off during the adhesion measurement. The coating prepared using only RP as the flame retardant exhibited Grade 3 adhesion, which is considered good, exhibited no cracks after the 100 cm drop hammer test, and an LOI of only 26.40%. The coating with 20 wt.% RP exhibited an LOI of only 26.4%, and that with the maximum content of 30 wt.% exhibited an LOI of 35.10%. However, because the viscosity of the mixed emulsion was extremely large, the surface of the coating with 30 wt.% RP contained air bubbles, and the film-forming property was poor. Table 2 shows the test results.

**Figure 3:**
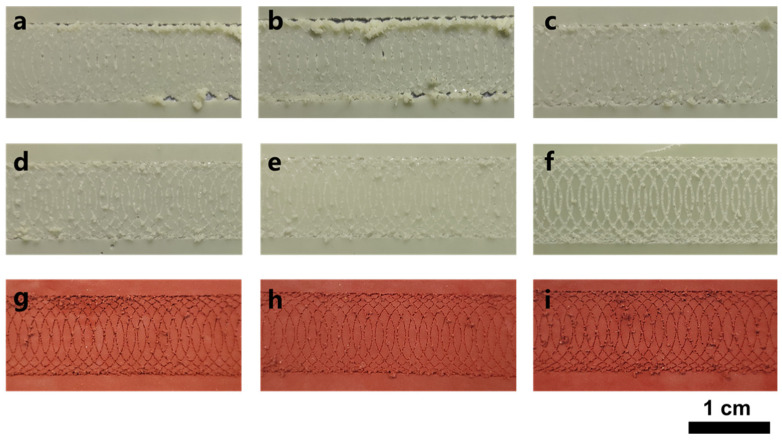
The change in the coating adhesion when samples were dried at room temperature and under different drying times. ATH 20 wt.% drying time: (**a**) 7 days, (**b**) 14 days, (**c**) 21 days, (**d**) 28 days, (**e**) 35 days, (**f**) 90 days; RP 20 wt.% drying time: (**g**) 3 days, (**h**) 6 days, (**i**) 14 days.

The maximum RP and ATH concentrations that could be added were 30 and 50 wt.%, respectively (Figure 1). The possible reason for the difference in the adhesion properties of the two flame retardants is the differences in the water contents of the emulsions prepared using the two flame retardants. Microencapsulated RP is wrapped in melamine resin. The coating material can effectively reduce the moisture absorption rate of RP, so that RP occurs in a dry state. The film formed from the RP-based flame-retardant emulsion through the DIFS method has a low water content. ATH contains crystal water, and the ATH-based film has a high water content. To prove this speculation, films were formed through DIFS using the same mass concentrations of RP- and ATH-based emulsions. The products were placed at room temperature under the same environmental conditions, and the relationship between the adhesion performance and the drying time of the flame-retardant coating was determined. Over time, the adhesion performance of the ATH flame-retardant coating gradually increased, and the highest grade reached Grade 3, after 35 days (Figure 3a–f). The RP flame-retardant coating exhibited Grade 3 adhesion after 6–14 days (Figure 3g–i). The difference in the short-term adhesion properties of the two flame-retardant coatings was due to the difference in water content [27]; the 906 emulsion was selected as the matrix, so that the flame retardant coating has adhesive performance, and lower water content will bring better short-term adhesion.

### 3.4. Two-Component Mixing of RP and ATH

The ATH-based coating exhibited good flame retardancy properties, but a low short-term adhesion performance and considerably improved adhesion after drying over a long time. The process was time-consuming, and RP exhibited a lower LOI value but stronger short-term adhesion performance than ATH. Therefore, to combine the advantages of the mechanical properties and flame retardancy properties of RP and ATH, we mixed the two flame retardants and added the mixture to the emulsion. Moreover, we explored the effects of the mixing ratio of 906 emulsion, ATH, and RP on the film properties.

**Figure 4:**
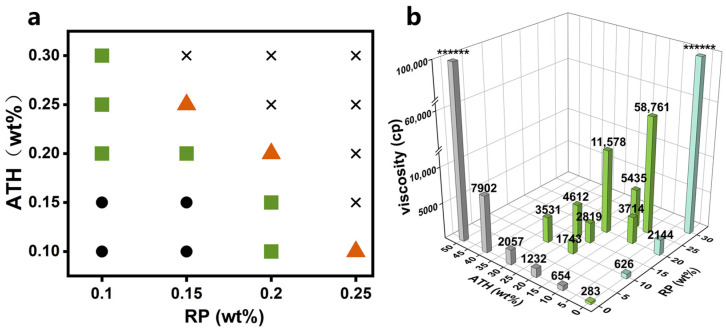
(**a**) The green square represents the range of emulsions with good film-forming properties; the orange triangle represents the mixed emulsions with cracks on the film surface; the cross represents the emulsions that are speculated to be unable of forming a good film; the circle represents the emulsions that are speculated to be able to form a good film; (**b**) Viscosities of the emulsions with different flame retardant concentrations. Gray represents the emulsion viscosity after mixing ATH alone into 906 emulsion, green represents the emulsion viscosity after mixing RP and ATH into 906 emulsion, blue represents the emulsion viscosity after mixing RP alone into 906 emulsion.

First, according to the morphology of the flame-retardant coatings prepared with different addition ratios, we selected the mixing ratio range that could prepare a good coating. In Figure 4a, the green square represents the mixing ratio with which a good coating can be prepared through the DIFS method; the orange triangle represents the mixing ratio at which a good coating cannot be formed, and cracks will appear after the surface dries; the black cross represents the speculated mixing ratio at which a good coating cannot be formed, and the black circle represents the speculated mixing ratio with which a good coating can be formed.

At some mixing ratios, the bubbles introduced during the stirring and mixing process could not be self-eliminated in the mixed emulsion with high viscosity (Figure 4a). To eliminate the bubbles, it was necessary to pump down to produce negative pressure to 0.9 MPa in a sealed container for over 8 h, so that the surface morphology of the flame-retardant coating was not affected. To accelerate the mixing process of the emulsion and reduce the mixing time, the viscosities of the emulsions with feasible mixing ratios were measured using a rotational viscometer, and the emulsion viscosities of individual flame retardants RP and ATH after mixing were also measured. Figure 4b shows the viscosities of different emulsion systems. Among the mixed emulsions, the ATH 20 wt.% + RP 10 wt.% emulsion exhibited the lowest viscosity (1743 pc). In the actual situation, the above-mixed emulsion did not need to be pumped down. The emulsions were allowed to stand for 4 h after mixing to eliminate the bubbles, and the coating surface was smooth. Considering that the negative pressure condition is not suitable for a large-scale operation and that additional processes will be added, the low-viscosity two-component emulsion system that did not require pumping down was selected. Because of the high viscosity, the maximum addition amounts of RP (30 wt.%) and ATH (50 wt.%) exceeded the measuring range of the instrument by 100,000 pc and thus were replaced by the symbol ******.

**Figure 5:**
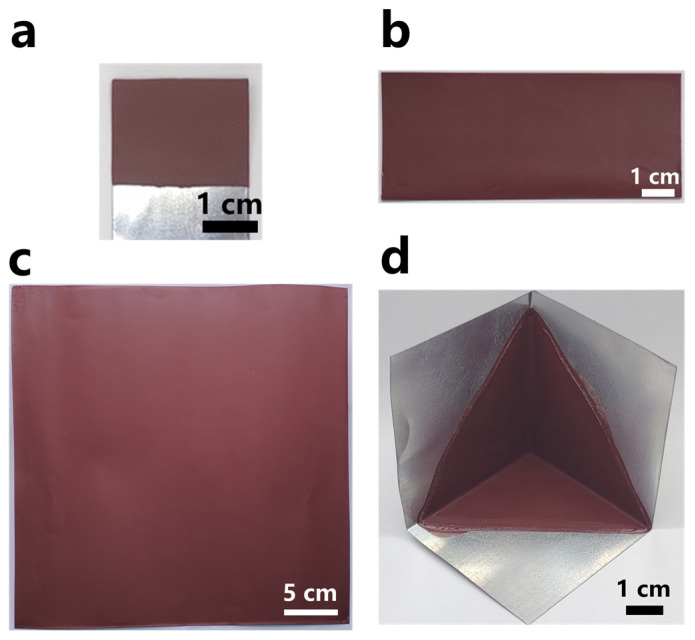
Film formed using ATH 20 wt.% + RP 10 wt.% mixed emulsion (**a–c**); planar film formed on areas of different sizes; (**d**) film formed at non-planar corners.

Flame-retardant coatings of different sizes and surface shapes were prepared via the DIFS method using the emulsion with the mixing ratio of ATH 20 wt.% + RP 10 wt.% (Figure 5).

### 3.5. Performance Test of The Mixed System of ATH 20 wt.% and Microencapsulated RP 10 wt.%

**Figure 6:**
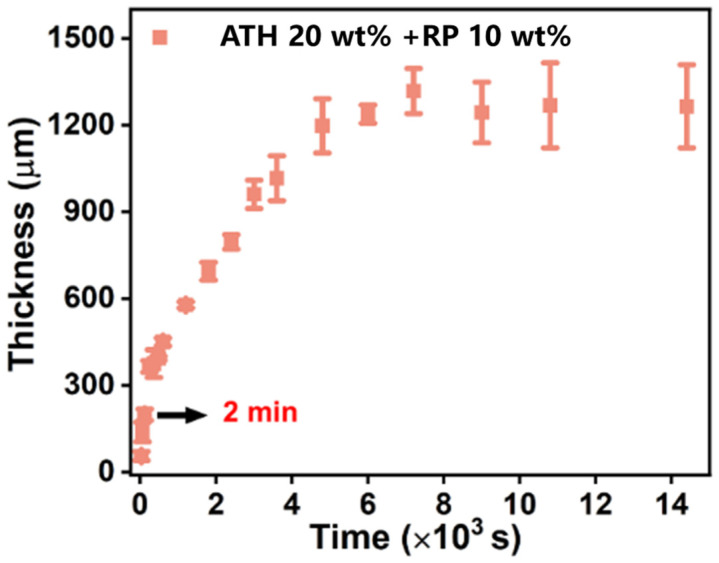
Growth curves of RP 10 wt.% and ATH 20 wt.%.

**Figure 7:**
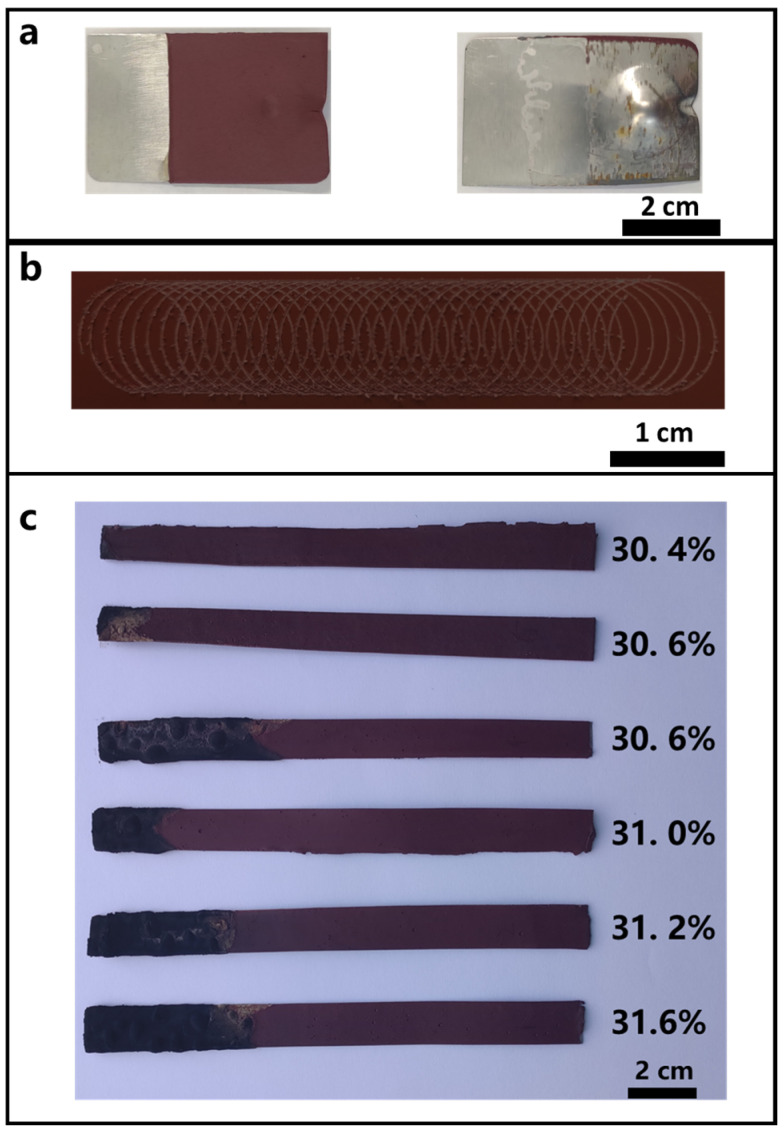
Test of the flame-retardant coating with a mixing ratio of RP 10 wt.% and ATH 20 wt.%: (**a**) 100 cm drop hammer anti-impact performance test; (**b**) adhesion performance test; (**c**) determination of LOI value.

Similar to the film thickness of the single-component flame-retardant coating in Figure 2, the mixed emulsion under the selected conditions had a relatively fast film-forming speed (Figure 6), and a coating with the desired thickness could be prepared within a short time. The coating of this mixing ratio possessed good mechanical properties, combining the good flame retardancy properties of ATH and the good adhesion properties of RP (Table 3). After multiple tests, as shown in Figure 7, the LOI value was 30.4%.

The flame-retardant layer was prepared through the DIFS method because a coating with a uniform thickness could be formed on the complex metal surface and the surface of the large substrate. Therefore, the flame-retardant coating was prepared on the corner of common metal components with a surface area of 30 cm × 30 cm (Figure 5c,d).

### 3.6. Preparation of Large-Area Flame-Retardant Coating

**Figure 8:**
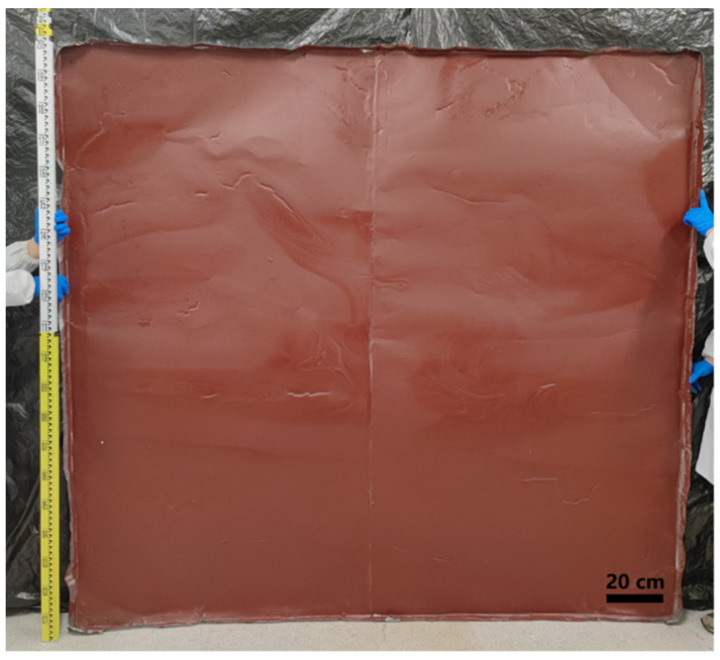
Image of the actual 2 m × 2.2 m flame-retardant coating.

After determination of the mixing ratio of flame retardants and the film formation test of metal sheets of different shapes and areas, to reduce the cost of large-scale industrial applications and the difficulty of obtaining raw materials, galvanized iron sheets, instead of zinc sheets, were used to prepare flame-retardant coatings. Two 1.2 m × 2 m metal sheets were spliced to prepare a large-area galvanized iron sheet, and the flame-retardant coating was prepared via the DIFS method. A 2 m × 2.2 m large-area flame-retardant coating was prepared, as shown in Figure 8.

## 4. Conclusions

In this experiment, a flame-retardant coating was prepared using a novel DIFS method. The materials used included an RE–906 emulsion, ATH and RP. Exploring the performance of the coating after mixing ATH and RP in RE–906 emulsion alone, it was found that a coating with a flat surface morphology can be achieved with 40 wt.% content of ATH and 20 wt.% content of RP. When this limit value is exceeded, there will be high viscosity, which causes the material failure to form a coating with a flat surface morphology. In this range, when the LOI value of ATH is higher, the adhesion performance is poor; similarly, when the LOI value of RP is lower, the adhesion performance is good. The final choice, therefore, is to use both ATH and RP in appropriate proportions. Hence, the effect of different mixing ratios of ATH and RP on viscosity was explored, and the optimum mixing ratio was determined. Finally, 20 wt.% ATH and 10 wt.% microencapsulated RP were found to be the optimum contents to be added to the RE–906 PUA emulsion. The mixture was mixed evenly, and the flame-retardant coating was prepared on the metal surface via the DIFS method. The adhesion, impact resistance, and LOI of the prepared metal coating sample were tested according to the GB 1720–1979, GB 1732–1993, and GBT2406.2–2009 standards, respectively. The adhesion of the flame-retardant coating with the selected mixing ratio reached the Grade 3 standard, the sample remained intact after the 100 cm drop hammer test, and the LOI reached 30.4%. Metal surface coatings with good flame retardancy properties were successfully prepared.

In practical application scenarios, the mainstream coating methods include brushing, roll coating, scraping and spraying [8,9]. Compared with the above-mentioned conventional preparation methods, the DIFS method can achieve accurate control of thickness and uniform coating on plane or non-plane surfaces. The conventional method is to simply apply the coating on the surface of the material and wait for the volatilization and drying of the solvent. Aqueous-phase coating, which is environmentally friendly, has a large latent heat of evaporation and hence, a long drying time [12]. Conversely, the organic phase coating with a short drying time pollutes the environment [13]. However, the DIFS method can perfectly combine the environmental safety of the aqueous phase coating with short drying time seen in organic phase coating because its film-forming mechanism is that the ions deposit the emulsion onto the surface of substrates only after demulsification. Electrical equipment generally contains a metal shell and has requirements for flame-retardant performance [6]. The DIFS method can meet such needs in electrical appliances. Materials with good flame retardant effects can be obtained in the existing research on flame retardant materials [1,2,3,4,5,6,7,14,15,16,17,18,19,20,21,22,23], but conventional coating preparation methods are still adopted. Herein, this article focused on the use of DIFS method. Using this method and combining different types of emulsions with inorganic metal flame retardants, intumescent flame retardants, brominated flame retardants, carbon nanotubes, graphene oxide and other flame retardant materials to prepare different types of flame retardant coatings, it is believed that better flame retardant coatings can be produced.

In summary, the DIFS method, used to successfully prepare flame retardant coatings, is environmentally friendly and nonpolluting. The use of water-based paints for the preparation favors environmental protection, as the paints have lower VOC concentration than in organic paints. Moreover, the ion demulsification process of the DIFS method accelerates the solidification of water-based paint and shortens the application period. The controllable flame-retardant coatings prepared via DIFS method in this study have potential applications in related fields.

## Figures and Tables

**Table 1:** Models of the instruments used in the experiment.

Instrument	Model	Manufacturer	City/ Country of Origin
Limiting oxygen index test apparatus	LX–4328	AISRY	Dongguan/China
Paint film impact tester	QCJ–100	Tianjin Kexin	Hangzhou/China
Electric paint film adhesion tester	QFD	Tianjin Shibo Weiye Chemical Glass Instrument	Guangzhou/China
Rotational viscometer	NDJ–5S	Shanghai Fangrui Instrument	Shanghai /China

**Table 2:** Mechanical properties and oxygen index of one-component mixed emulsions.

Flame Retardant	wt.%	Adhesion Grade	Impact (cm)	LOI (%)
ATH	10	×*	100	--*
ATH	20	×	100	29.90
ATH	30	×	100	--
ATH	40	×	100	39.30
ATH	50	×	100	--
RP	10	3	100	--
RP	20	3	100	26.40
RP	30	3	100	35.10

×* means the adhesion is too low to be measured; --* means the data is not tested.

**Table 3:** Performance test of the RP 10 wt.% and ATH 20 wt.% mixed system.

Electrode Material	Adhesion	Impact	LOI
Zinc sheet	Grade 3	100 cm	30.4%
Galvanized iron sheet	Grade 3	100 cm	

## Data Availability

Not applicable.

## Supplementary Materials

The following supporting information can be downloaded at: https://www.mdpi.com/article/10.3390/polym15030754/s1, Figure S1: (a) The flame-retardant coating sample for testing the anti-impact performance; (b) The flame-retardant coating sample for testing adhesion performance; (c) The sample for testing the limiting oxygen index.
