# Supplementary material for: Rapid Preparation of Flame-Retardant Coatings Using Polyurethane Emulsion Mixed with Inorganic Fillers
Source: Polymers (Basel). 2023 Feb 2;15(3):754. doi: 10.3390/polym15030754 (PMC9919632; doi:10.3390/polym15030754)
Supplement: Supplementary file 1 [file polymers-15-00754-s001.zip › polymers-2145415-supplementary.pdf]

Supporting Information

The schematic diagram of the results of sample performance that was tested according to the standards and specifications. Figure S1a shows the sample after it was tested using the paint film impact tester according to the standard GB 1732–1993. Figure S1b shows the sample after the electric paint film adhesion tester was used to characterize the adhesion performance of the coating according to the standard GB 1720–1979. Figure S1c shows the sample after the limiting oxygen index (LOI) of the material was determined according to the standard GBT2406.2–2009.

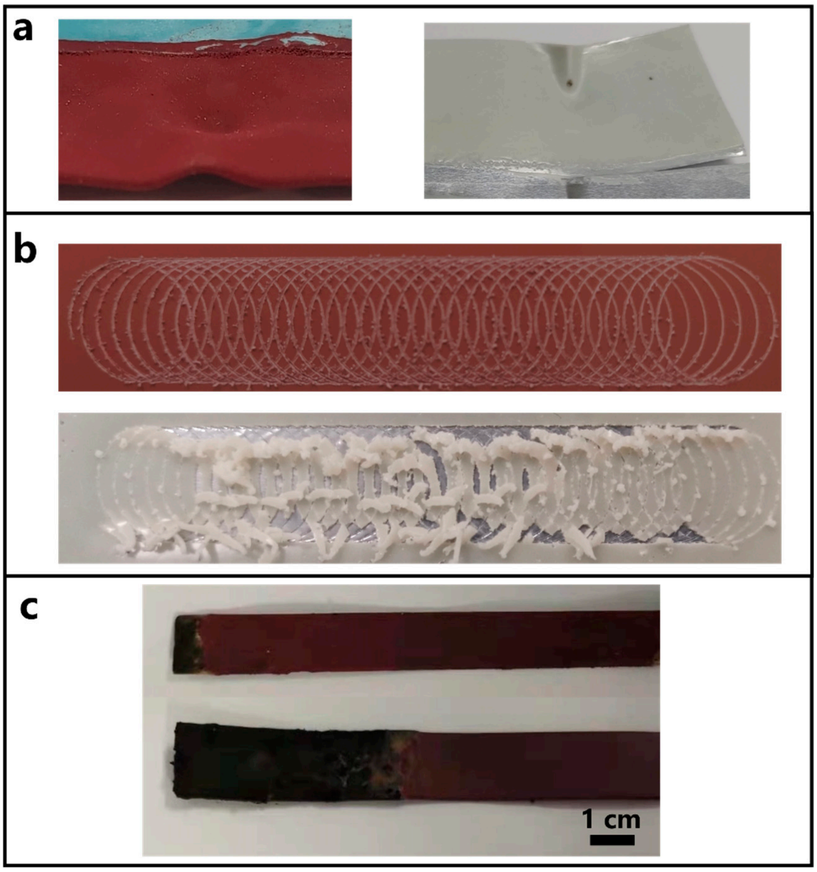

Figure S1. (**a**) The flame-retardant coating sample for testing the anti-impact performance; (**b**) The flame-retardant coating sample for testing adhesion performance; (**c**) The sample for testing the limiting oxygen index.
